# Antimicrobial resistance: a priority for global health action

**DOI:** 10.2471/BLT.15.158998

**Published:** 2015-07-01

**Authors:** Arthur Chioro, Awa Marie Coll-Seck, Bent Høie, Nila Moeloek, Aaron Motsoaledi, Rajata Rajatanavin, Marisol Touraine

**Affiliations:** a Ministry of Health, Brasilia, Brazil.; b Ministry of Health and Social Affairs, Dakar, Senegal.; c Ministry of Health and Care Services, Teatergata 9, Postboks 8011 Dep, 0030 Oslo, Norway.; d Ministry of Health, Jakarta, Indonesia.; e Ministry of Health, Pretoria, South Africa.; f Ministry of Public Health, Bangkok, Thailand.; g Ministry of Social Affairs, Health and Women’s Rights, Paris, France.

From the earliest days of antibiotics, scientists have warned that without careful management of the way these powerful drugs are used, pathogens could quickly develop defences against them. Today, antimicrobial resistance is spreading faster than ever, threatening the effectiveness of many of our most important weapons against infections.^1^

Convened by the Foreign Policy and Global Health Initiative group of nations,^2^ experts met in Oslo, Norway in November 2014 to consider ways to reverse the dangerous spread of resistance to antibiotics and other antimicrobial medicines. As ministers charged with safeguarding the health of the public, we view this as a top priority.

In an increasingly interconnected world, the challenge of combating antimicrobial resistance cannot be addressed by individual countries acting alone. We have therefore actively supported efforts by the World Health Organization (WHO) to develop a global action plan to combat antimicrobial resistance.^3^

While antimicrobial medicines also play an important role in agriculture,^4^ their essential function is to protect human health. Our focus is on ways to improve the stewardship of these critical medicines so they will maintain their usefulness.

We recognize the burden faced by those living in poverty in low- and middle-income countries, who have limited access to these medicines, often at prices they cannot afford, forcing them to skimp on treatment courses. This puts them at particular risk from infections. Accordingly, we must simultaneously address issues of access along with improved stewardship and strengthened policies for the rational use of antimicrobial medicines.

The Oslo meeting recommended that: everyone should have access to appropriate, effective and affordable antimicrobial medicines in a timely manner; the use of antimicrobials needs to be well regulated and based on medical need and appropriate diagnosis; inappropriate, unnecessary and dangerous use of these medicines must be actively discouraged; manufacturers and marketers of these medicines should become active partners in furthering appropriate use and stopping excessive and inappropriate use; the direct marketing to consumers of antimicrobial medicines by manufacturers, importers and distributors should be forbidden or tightly controlled.^5^

We are committed to combating the scourge of low-quality antimicrobial products, which risks poor health outcomes for their users as well as the spread of antimicrobial resistance. WHO has a role as the platform for discussions on this problem in the interest of public health, in particular through the SSFFC (substandard/spurious/falsely-labelled/falsified/counterfeit medical products) Member State mechanism. We will work to strengthen our health systems to accelerate the implementation of proven infection prevention and control measures that will reduce the need for antimicrobials. We will also work with other sectors – particularly agriculture, animal husbandry and manufacturing – to ensure that their use of antimicrobial agents is controlled and monitored, to end inappropriate use, prevent the contamination of food and the environment and limit the development of resistance. Certain new classes of antimicrobials, once they are developed, should be restricted to human use only and under tightly controlled conditions.

Countries with limited resources and vulnerable health systems need particular help from the international community to strengthen infection prevention and control, as well as the stewardship of antimicrobial medicines. Development of sustained investments in health systems must be a common commitment. Through WHO and others, we will work together with countries that need assistance to strengthen their own capacities and systems – in particular, to meet the requirements of the International Health Regulations.^6^

Antimicrobial medicines are among humankind’s greatest scientific discoveries. They make us all safer. It is our responsibility and our pledge to rationally manage and preserve this vital resource for generations to come.
